# Holding Multiple Items in Short Term Memory: A Neural Mechanism

**DOI:** 10.1371/journal.pone.0061078

**Published:** 2013-04-16

**Authors:** Edmund T. Rolls, Laura Dempere-Marco, Gustavo Deco

**Affiliations:** 1 Oxford Centre for Computational Neuroscience, Oxford, United Kingdom; 2 Department of Information and Communication Technologies, Universitat Pompeu Fabra, Barcelona, Spain; 3 Institució Catalana de Recerca i Estudis Avançats (ICREA), Barcelona, Spain; Duke University Medical Center, United States of America

## Abstract

Human short term memory has a capacity of several items maintained simultaneously. We show how the number of short term memory representations that an attractor network modeling a cortical local network can simultaneously maintain active is increased by using synaptic facilitation of the type found in the prefrontal cortex. We have been able to maintain 9 short term memories active simultaneously in integrate-and-fire simulations where the proportion of neurons in each population, the sparseness, is 0.1, and have confirmed the stability of such a system with mean field analyses. Without synaptic facilitation the system can maintain many fewer memories active in the same network. The system operates because of the effectively increased synaptic strengths formed by the synaptic facilitation just for those pools to which the cue is applied, and then maintenance of this synaptic facilitation in just those pools when the cue is removed by the continuing neuronal firing in those pools. The findings have implications for understanding how several items can be maintained simultaneously in short term memory, how this may be relevant to the implementation of language in the brain, and suggest new approaches to understanding and treating the decline in short term memory that can occur with normal aging.

## Introduction

Short term memory can be implemented in for example the prefrontal cortex by the continuing firing of neurons during the short term memory period [1]–[3]. Here, we show how synaptic facilitation in the recurrent synaptic connections can increase very considerably the number of short term memories that can be maintained simultaneously active in a standard model of this continuing firing, a cortical attractor network [4]–[7], and can make the short term memory system much more robust, i.e. less sensitive to the selection of model parameters such as the amount of inhibition. The findings are of importance for understanding how several different items can be maintained simultaneously in working memory [8], the impairments of cognitive function including working memory and attention in schizophrenia and in aging that can occur when the operation of cortical systems for short term memory and attention are impaired [7], [9]–[12], and how cortical areas involved in language processing can keep active several items, such as the subjects of a sentence [13].

George Miller published a paper in 1956 entitled “The magic number 7, plus or minus two: some limits on our capacity for processing information” [14]. A key issue was that the capacity of short term memory (for example our memory for a list of telephone numbers) is approximately 7±2 items. For visual short term memory, the capacity may be closer to 4 items [8], [15], [16].

Kohonen and other pioneers made neuronal network models that could maintain their activity during a short term memory period [17]–[19]. A key to the operation of such autoassociation networks was the strong recurrent synaptic connectivity within each population of excitatory neurons, and feedback inhibition to all the excitatory neurons to maintain the activity within limits, and just some populations firing actively. Hopfield introduced the methods of statistical mechanics from theoretical physics to allow for example the calculation of the capacity of what became known as attractor networks [4], [20], that was extended to more biologically plausible networks with diluted connectivity, sparse representations, graded firing rate representations, and the speed of operation of dynamically realistic integrate-and-fire neuronal attractor networks [6], [21]–[24].

To provide background information, we note that there are currently a number of theories regarding the underlying mechanisms that yield limits to the capacity of short term memory. The main two competing models are “fixed capacity models” (or slot models) [25], and dynamic allocation models (or resource models) [26]. In fixed capacity models, all items are recalled with equal precision up to the limit (3–4 items) with no further information being stored beyond this limit, whereas in dynamic allocation models, the limited resources are shared out between items but not necessarily equally. This is an issue that has recently received substantial attention (e.g. Wei et al. [27]), and our work does not directly tackle this question, although we suggest that with respect to formal network models, this issue arises with continuous attractor networks. Instead, we focus on another fundamental aspect and investigate the biophysical mechanisms that establish such capacity limits in discrete attractor networks, and how this short term memory capacity can be enhanced. Indeed, the issue arises of the extent to which discrete (as contrasted with continuous [28]) attractor networks with distinct subsets of neurons for each memory are able to maintain more than one memory simultaneously active. One memory at a time is typical, and it was shown that with overlapping memory patterns, more than 1–2 simultaneously active memories are difficult due to interference between the patterns even when the patterns are graded [29]. Most investigations have involved non-overlapping patterns as we do here, and it has been found difficult to maintain more than approximately 4 memories simultaneously active [30], [31]. Non-overlapping patterns refers to patterns in which each population of neurons representing a memory is separate, and this has advantages in the mean field analysis and the simulations. However, interference and cross-talk between the different pools was a feature of the simulations described here, and was implemented through *w_−_* as shown below. Nonetheless, by using sparse representations with *a* = 0.01 (where the sparseness *a* is the proportion of neurons that is active for any one memory pattern), Amit et al. were able to maintain six memory patterns simultaneously active in short term memory [32], and mean field analysis showed that having reasonable number of patterns simultaneously active could be stable with sparse representations [30], [32]. Here, we show how the addition of synaptic facilitation to this approach can increase very considerably the number of short term memories that can be maintained simultaneously active, and can make the short term memory system much more robust.

## Methods

**Figure 1:**
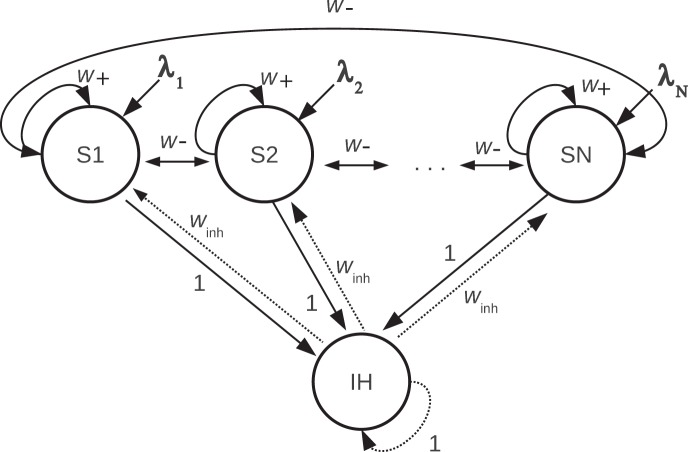
The attractor network model. The single network is fully connected. The excitatory neurons are divided into N selective pools or neuronal populations S1–SN of which three are shown, S1, S2 and SN. There were typically N = 10 short term memory populations of neurons in the integrate-and-fire networks simulated, and analyzed with mean field analyses. Each of these excitatory pools represents one short term memory by maintaining its activity during a delay period after a cue has been applied. We show that if the excitatory connections show synaptic facilitation, then any number in the range 0–9 of the pools will maintain its activity in the delay period depending on which set of pools was activated by a cue λ_1_, λ_2_, … λ_10_. The synaptic connections have strengths that are consistent with associative learning. In particular, there are strong intra-pool connection strengths *w*_+_, and weaker between-pool synaptic connection strengths of *w*_−_. The excitatory neurons receive inputs from the inhibitory neurons with synaptic connection strength *w*_inh_. The other connection strengths are 1. *w*_+_ was typically 2.3, *w*_−_ 0.87, and *w*_inh_ 0.945 as determined using a modified mean field analysis. The integrate-and-fire spiking network typically contained 1000 neurons, with 80 in each of the 10 non-overlapping short term memory (STM) excitatory pools, and 200 in the inhibitory pool. Each neuron in the network also receives external Poisson inputs λ_ext_ from 800 external neurons at a typical rate of 3.05 Hz/synapse to simulate the effect of inputs coming from other brain areas.

The attractor network architecture for short term memory that was simulated is shown in Fig. 1. The network investigated had 10 excitatory pools of neurons, S1–S10, and one inhibitory pool of neurons, with the synaptic weights shown in Fig. 1. The global inhibition in the model reflects the evidence that in a local area of the cerebral cortex, inhibitory neurons are connected to excitatory neurons within that local area [33]. In the network, the proportion of neurons active for any one representation, that is the sparseness with binary neurons, was 0.1, and this value was chosen as this is in the order of the sparseness of the representation in the cerebral cortex [34]. A full description of the integrate-and-fire attractor single network is provided below in subsections ‘Network’ and ‘Spiking Dynamics’. The network has a mean field equivalent allowing further quantitative analysis of the stability conditions and capacity, and the way in which we used this in the context of synaptic facilitation is described in subsection ‘Mean field analysis’.

To the standard integrate-and-fire network [7], [35]–[37], we added synaptic facilitation, which has been incorporated into such networks, for example to account for the low firing rates in the delay periods of some short term memory tasks [38], in decision tasks with responses delayed over a period in which the firing rate may be low [39], and decision-making with sequentially presented stimuli [40]. In contrast to Mongillo et al.’s work [38], we investigate the neurodynamical origin of short term memory capacity limits, and assume that sustained neural activation (as opposed to alternative mechanisms such as neural oscillations and/or patterns of synaptic strengths already reviewed by the authors elsewhere [31]) is the mechanism underlying the encoding and maintenance of multiple items in short-term memory.

Synaptic facilitation is common in higher cortical areas such as the prefrontal cortex [41]–[43] implicated in working memory and attention [2], [8]. Synaptic facilitation is caused for example by the increased accumulation of residual calcium at the presynaptic terminals, which increases the probability of neurotransmitter release [41]. Short term synaptic facilitation was implemented using a phenomenological model of calcium-mediated transmission [38]. The synaptic efficacy of the recurrent connections between all of the excitatory neurons was modulated by the utilization parameter *u* (the fraction of resources used) reflecting the calcium level. When a spike reaches the presynaptic terminal, calcium influx in the presynaptic terminal causes an increase of *u* which increases the release probability of transmitter and thus the strength of that synapse. The time constant of the decay of the synaptic facilitation is regulated by a parameter *τ*_F_ which experimentally is around 1–2 s [38], [43]. The value for the baseline utilization factor *U* (0.15) and for τ_F_ (1.5 s) used here are similar to values reported experimentally and used elsewhere [38]–[40], [43]. In more detail, the strength of each recurrent excitatory synapse *j* is multiplied by the presynaptic utilization factor *u_j_* (*t*), which is described by the following dynamics:
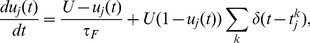
(1)where 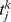 is the time of the corresponding presynaptic spike *k*. The first term shows how the synaptic utilization factor *u_j_* decays to the baseline utilization factor *U* = 0.15 with time constant *τ_F_* = 1500 ms, and the second term shows how *u_j_* is increased by each presynaptic action potential *k* to reach a maximum value of 1 when the neuron is firing fast. The modulation by the presynaptic utilization factor *u* is implemented by multiplying the synaptic weight by *u* to produce an effective synaptic weight *w*_eff_. This models the underlying synaptic processes [38].

### Network

The integrate-and-fire attractor network, developed from an earlier model [7], [35]–[37] but with synaptic facilitation added [38], contains *N_E_* = 800 pyramidal cells (excitatory) and *N_I_* = 200 interneurons (inhibitory). There are ten populations or pools of excitatory neurons each with 0.1 *N_E_* neurons (i.e. 80 neurons). The network is fully connected. Neurons within the selective population are coupled, by a factor *w*_+_ = 2.3 (unless otherwise stated) above the baseline connection synaptic weight *w* = 1. Connections to inhibitory cells are set to the baseline level, *w = *1, and from the inhibitory neurons *w*_inh_ = 0.945 (unless otherwise stated).

To model spontaneous background activity, every neuron in the network is coupled through *N*_ext_ = 800 synaptic connections to an external source of Poisson-distributed, independent spike trains of rate 3.05 Hz per synapse, so that each neuron received 2440 spikes/s. The presence of cue stimuli to initiate the short term memory was modeled by an increase of *λ* to 3.3125 spikes/synapse. This value of *λ* was applied to any of the pools S1 to S10 (via *λ*_1_ to *λ*_10_ as illustrated in Fig. 1 of the paper) during the cue delivery period, which was from 500–1500 ms in the simulations. For pools not being cued on, and for the inhibitory neurons, *λ* remained at *λ*_ext_ during the cue delivery period and throughout the trial. After the cue had been delivered to the pools selected for short term memory for that trial, *λ* returned to the default value of *λ*_ext_ = 3.05 Hz/synapse for the remainder of the trial (1500–4500 ms).

### Spiking Dynamics

The model is based on integrate-and-fire (IF) neurons. The subthreshold membrane potential *V* of a neuron evolves according to
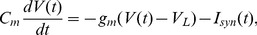
(2)where *C_m_* is the membrane capacitance (see numerical values in Table 1), *g_m_* is the membrane leak conductance, *V_L_* is the resting potential, and *I*_syn_ is the synaptic current.

**Table 1:** Parameters used in the integrate-and-fire simulations.

*C_m_* (excitatory)	0.5 nF
*C_m_* (inhibitory)	0.2 nF
*g_m_* (excitatory)	25 nS
*g_m_* (inhibitory)	20 nS
*V_L_*	−70 mV
*V*_thr_	−50 mV
*V*_reset_	−55 mV
*V*_E_	0 mV
*V*_I_	−70 mV
*g_AMPA,ext_* (excitatory)	2.08 nS
*g_AMPA,rec_* (excitatory)	0.104 nS
*g_NMDA_* (excitatory)	0.327 nS
*g_GABA_* (excitatory)	1.25 nS
*g_AMPA,ext_* (inhibitory)	1.62 nS
*g_AMPA,rec_* (inhibitory)	0.081 nS
*g_NMDA_* (inhibitory)	0.258 nS
*g_GABA_* (inhibitory)	0.973 nS
*τ_NMDA, decay_*	100 ms
*τ_NMDA,rise_*	2 ms
*τ_AMPA_*	2 ms
*τ_GABA_*	10 ms
*α*	0.5 ms^−1^

The synaptic current includes glutamatergic excitatory components (mediated by AMPA and NMDA receptors) and inhibitory components (mediated by GABA). External cells contribute to the current only through AMPA receptors. The total current is given by
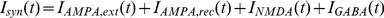
(3)with the different currents defined by



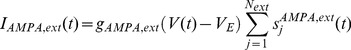
(4)

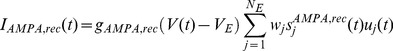
(5)

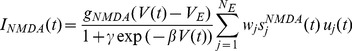
(6)

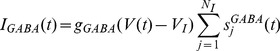
(7)where 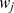 are the synaptic weights, 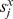 is the fraction of open channels for each receptor, and 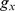 is the synaptic conductance for receptor *x* = AMPA, NMDA, GABA. Synaptic facilitation is implemented through the utilization factor *u_j_* which modulates the recurrent excitatory synapses, specifically the synaptic gating variables *s* as can be seen from Equations 5 and 6. The values for the synaptic conductances and the reversal potentials *V_E_* and *V_I_* are given in Table 1. NMDA currents are voltage dependent and controlled by the intracellular magnesium concentration ([Mg^2+^] = 1 mM), with parameters *γ* = [Mg^2+^]/(3.57 mM) = 0.280 and *β = *0.062 (mV) ^−1^.

The fraction of open channels in cell *j*, for all receptors, is described by the following differential equations, where 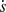 is the derivative of *s* in time:
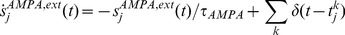
(8)

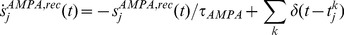
(9)

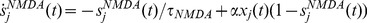
(10)

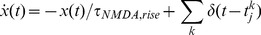
(11)

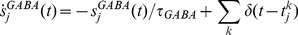
(12)where the rise time constant for NMDA currents is *τ_NMDA,rise_* = 2 ms, and α* = *0.5 ms^−1^; rise time constants for AMPA and GABA currents are neglected. Decay time constants for AMPA, NMDA, and GABA synapses are *τ_AMPA_* = 2 ms, *τ_NMDA,decay_* = 100 ms, and *τ_GABA_* = 10 ms. The sums over *k* represent a sum over spikes emitted by pre-synaptic neuron *j* at time 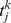.

### Mean Field Analysis

To complement the integrate-and-fire simulations described in the paper, we performed mean field analyses of the network when it is operating with synaptic facilitation. The mean field analysis provides a simplification of the integrate-and-fire dynamics considered in the spiking simulations, which are computationally expensive and therefore not suitable for extensive parameter explorations, though they are important for establishing how the system operates with stochastic dynamics when it is of finite size and has noise, that is randomness, introduced by the almost random spiking times of the neurons for a given mean rate [7]. The mean field analyses allow the different operating regimes of the network to be established analytically [7], [44], [45], including in our case proving the stability of the system when multiple short term memories are simultaneously active. The mean field analyses also allow exhaustive parameter explorations to define the values of the parameters within which different operating regimes of the network occur. The mean field analysis is noiseless, that is there is no randomness introduced by the almost random spiking times of the neurons for a given mean firing rate, and is in this respect equivalent to an infinitely large integrate-and-fire spiking simulation. The network that we simulated has, without the synaptic dynamics, a mean field equivalent that was developed by Brunel and Wang [35] (see Text S1 for a full account of the original method). Here, we consider that mean field approach but extend and apply it to the case where synaptic facilitation is present in the network. In the following we describe how the conventional mean field approach has been extended.

#### Synaptic facilitation in the mean field approach

**Figure 2:**
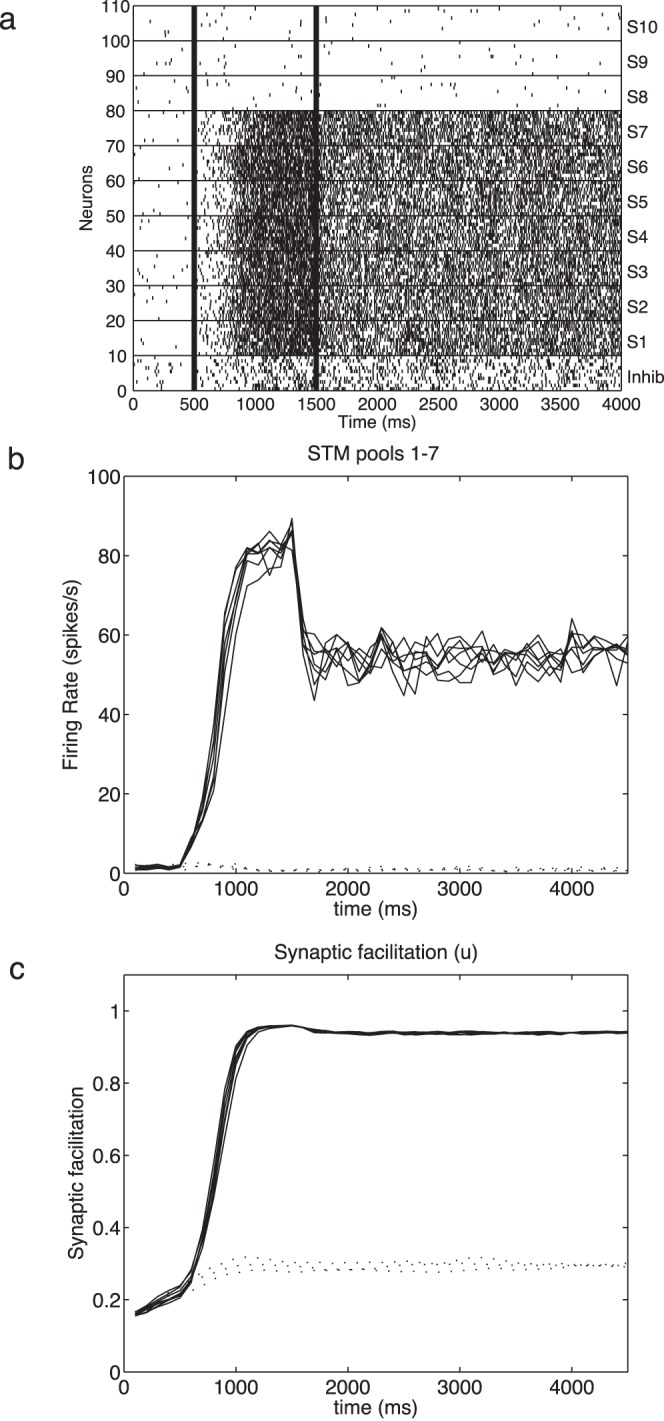
Short term memory with 7 simultaneously active memories. After a 0.5 s period of spontaneous activity (with λ_ext_ at the baseline level of 3.05 Hz/synapse), cues λ_1_−λ_7_ were applied to excitatory neuron pools S1 to S7 during the period 500–1500 ms. (λ_1_−λ_7_ were applied by increasing λ_ext_ to 3.3125 Hz/synapse for just these 7 pools during the cue period.) As shown on the rastergram (a) and peristimulus time histogram of the firing rate (b) this produced a high firing rate of approximately 70 spikes/s in each of the pools S1–S7 (b, solid lines), and the synaptic utilization factor *u_j_* increased in this period to values close to 1 (c, solid line). In the rastergram (a) each vertical line is a spike from a single neuron, and each row shows the spikes for a single neuron. 10 neurons chosen at random are shown for each pool of neurons. The input to pools λ_8_−λ_10_ remained at the baseline level of 3.05 Hz/synapse throughout the trial, and therefore their firing rates did not increase in the period 500–1500 ms (b, dashed lines), and correspondingly the utilization factor *u* for these pools remained low (c, dashed lines). At the end of the cue period, λ_1_−λ_7_ returned to the baseline level of 3.05 Hz/synapse, but in the short term memory period from 1500–4500 ms the neurons in pools 1–7 continued to fire at a high rate of approximately 40 spikes/s (b, solid lines), well above the baseline in the spontaneous period of 3 spikes/s. Moreover, the synaptic facilitation *u_j_* remained high for pools 1–7 during the short term memory period from 1500–4500 ms (c, solid lines).

We extended and modified the standard mean field approach [7], [35] to incorporate the effects of synaptic facilitation. As can be seen from Fig. 2c, the synaptic facilitation *u*(*t*) increases particularly for each excitatory subpopulation that has received external stimulation produced by the cues, and reaches a value close to 1 during the delay period. Only small increases occur in the pools that have not received this external stimulation by a memory cue (and those increases occur because of effects produced through the *w*_−_ connections between the excitatory pools shown in Fig. 1). Since *u*(*t*) converges in both cases to a given value, we can then define a new effective synaptic strength (*w*_j_)_eff_ which operates during the steady state, and this opens the possibility to perform a mean field analysis of the network endowed with STP. The effective synaptic strengths are defined as:
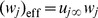
(13)where *u_j_*_∞_ is the asymptotic synaptic facilitation value that is estimated from the average *u_j_*(*t*) observed in the last 500 ms of the delay period of the spiking network simulations. The protocol used in such simulations is described in detail in the next section. A conventional mean field analysis with the effective synaptic strengths can then be performed and can be used to systematically scan the parameter space {*w_+_, w*_inh_} with the aim to reproduce a short term memory regime in which only the stimulated (i.e. cued) subpopulations remain active during the delay period.

## Results

### Integrate-and-fire Simulations

Results of the integrate-and-fire simulations of short term memory are shown in Fig. 2. After a 500 ms period of spontaneous activity, cues λ_1_−λ_7_ were applied to excitatory neuron pools S1 to S7 during the period 500–1500 ms. This produced a high firing rate of approximately 70 spikes/s in pools S1–S7 (Fig. 2b), and the synaptic utilization factor *u_j_* increased in this period to values close to 1 (Fig. 2c). The inputs λ_8_−λ_10_ to pools S8–S10 remained at the baseline level of 3.05 Hz/synapse throughout the trial, and therefore their firing rates did not increase in the period 500–1500 ms, and correspondingly the utilization factor *u* for these pools remained low. At the end of the cue period, λ_1_−λ_7_ returned to the baseline level of 3.05 Hz/synapse, but the neurons in pools 1–7 continued to fire at a high rate of approximately 40 spikes/s, well above the baseline rate of 3 spikes/s in the spontaneous period. The continuing relatively high firing rate in pools S1–S7 was sufficient to keep via the recurrent collateral connections the synaptic utilization factor within such pools relatively high (Fig. 2c), and that in turn was what kept each of the selective pools S1–S7 continuing to fire fast. That high firing rate in pools S1–S7 was the implementation of the short term memory. In contrast, the firing in the uncued pools S8–S10 remained low, showing that the short term memory was selective for just whichever pools of neurons had been cued on earlier (Fig. 2a,b). The inhibition prevented the effects of interference between the different neuronal pools implemented through *w*_−_producing high firing rates in the uncued neuronal pools.

In this scenario, the synaptic facilitation in the recurrent connections is regenerated by the continuing firing of the originally cued pools. Thus two factors are responsible for enabling the firing to continue for long periods of many seconds after the cues have been removed. The first is the recurrent collateral activity itself implemented in the architecture of the network. Second, it is the regenerating synaptic facilitation just for the pools that were cued and had high firing as a result, which gives the advantage in the competition implemented by the inhibitory neurons to the previously cued pools, relative to the previously uncued pools.

**Figure 3:**
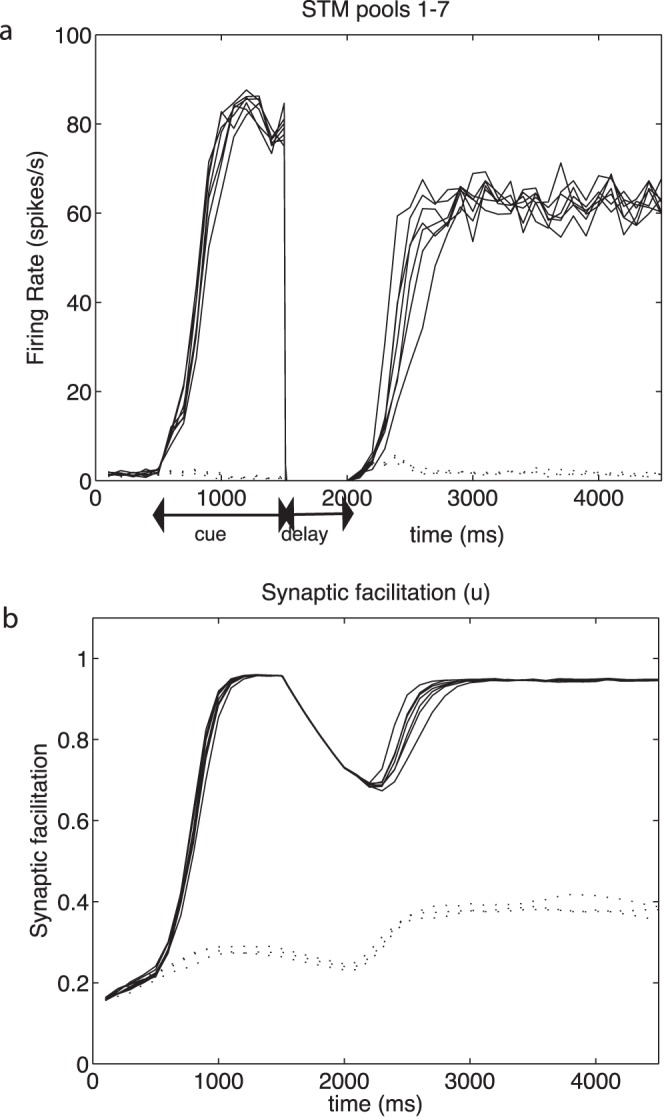
Multiple item short term memory can be selectively restored after a delay showing the importance of the synaptic facilitation in the short term memory process. After the cue period from 500–1500 ms, there was a delay period with λ_ext_ for the excitatory neurons set to 0 for 500 ms. When λ_ext_ was restored to all excitatory pools (in this case with a value of 3.125 Hz/synapse) at time *t* = 2000 ms, there was sufficient synaptic facilitation remaining (b) to produce firing selectively in the previously cued pools (a), and thus to restore the short term memory. Conventions as in Fig. 2. Without synaptic facilitation, the network failed to recover the previously cued memories.

Further evidence for the importance of the synaptic facilitation in the process is that if there was a short delay after the end of the cue period in the neuronal firing (produced in the simulations by a decrease of λ_ext_ to 0), then the firing in the previously cued pools could be restored by restoring λ_ext_ within approximately 1 s, before the synaptic facilitation had decayed too far. However, if λ_ext_ was delayed for longer (e.g. 3 s) so that the synaptic facilitation had decayed, then the firing of the previously cued pools could no longer be selectively restored by restoring λ_ext_ at its baseline or at any other value. An example is shown in Fig. 3, in which after the cue period from 500–1500 ms, there was a delay period with λ_ext_ for the excitatory neurons set to 0 for 500 ms. When λ_ext_ was restored to all excitatory pools (in this case with a value of 3.125 Hz/synapse) at time *t* = 2000 ms, there was sufficient synaptic facilitation remaining (Fig. 3b) to produce firing selectively in the previously cued pools (Fig. 3a), and thus to restore the short term memory. With delays of longer than approximately 2 s (depending on λ_ext_), the synaptic facilitation had dissipated so much that selective recall of the short term memories became very poor. This helps to show the importance of the synaptic facilitation in the maintenance and even restoration of in this case multiple short term memory representations.

**Figure 4:**
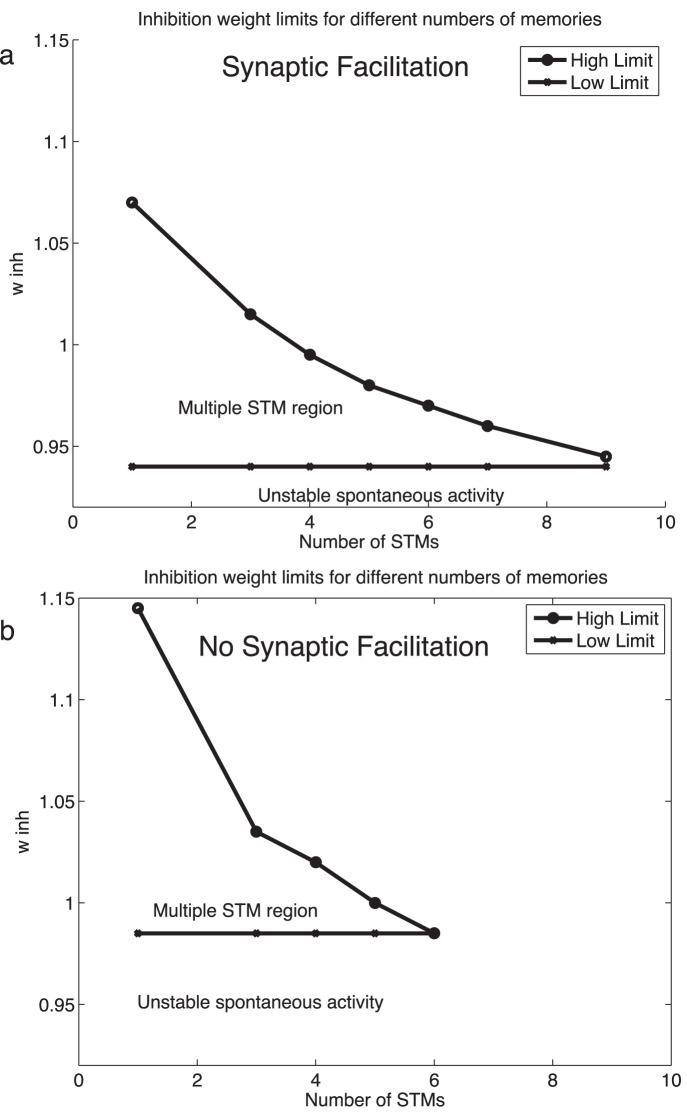
(a) The upper and lower values for the inhibitory synaptic weights in the network as a function of the number of memories that are simultaneously active. For example, the data plotted for the value 3 short term memories simultaneously active are the upper value that is possible in the network for 3 short term memories to be simultaneously active, and the lower value shows the lowest value of inhibition at which 0 memories can be active, that is, when the spontaneous firing state is stable without any cues being applied. The integrate-and-fire simulations were run with the standard value of *w*_+_ = 2.3, and all the other parameters at their standard values described elsewhere in the paper. The results are from the integrate-and-fire spiking simulations, and from the modified mean field analyses. (b) The same as (a), but without synaptic facilitation. The axes are to the same scale as in (a), but a slightly larger value of *w*_inh_ is needed to maintain stability of the spontaneous state as the effective *w*_+_ is now exactly 2.3, with no modulation by *u*.

In these simulations, with the parameters shown, we were able to keep any number between 0 and 9 of the pools of memory that represent each short term memory simultaneously active, depending on which pools were activated by the cue. The system described thus has the strength that for a fixed set of parameters, it can flexibly keep active any number of memories from 0–9. Without synaptic facilitation, we were able to maintain only 6 pools simultaneously active (*w*_+_ = 2.3, *w*_inh_ = 0.98) in the same network (Fig. 4b). Thus synaptic facilitation considerably increased the number of short term memories that could be maintained simultaneously from 6 to 9 with the sparseness *a* = 0.1. We note that the condition where the number of cued short term memory pools cued was 0 is an important condition that was satisfied in the results described, and shows that the network remained stably in its low firing rate spontaneous state when no pools were cued. The mean field analyses also confirmed this.

We investigated what parameters may be important in setting the limit on the number of short term memories that can be maintained active simultaneously. A key parameter was found to be the inhibitory synaptic weights in the model shown in Fig. 1. Fig. 4a shows the upper and lower values for the inhibitory synaptic weights in the network as a function of the number of memories that are simultaneously active. The data are from the integrate-and-fire simulations confirmed with the modified mean field analyses. For example, the data plotted for the value 3 short term memories simultaneously active are the upper value that is possible in the network for 3 short term memories to be simultaneously active, and the lower value shows the lowest value of inhibition at which 0 memories can be active, that is, when the spontaneous firing state is stable without any cues being applied. Lower values of *w*_inh_ than this resulted in the spontaneous state with no cues applied jumping into a high firing rate attractor state with rates typical of those found in the cerebral cortex [34]. The simulations were run with the standard value of *w*_+_ = 2.3, and all the other parameters at their standard values described elsewhere in the paper. The upper limit shown in Fig. 4a defines the level of inhibition above which the inhibition is too great for that number of short term memories to be simultaneously active. Clearly if more memories must be kept simultaneously active, the level of inhibition must not be too great, as otherwise some of the active attractors will be quenched. What Fig. 4a shows is that the upper limit as expected decreases as the number of memories required to be active increases, and that very interestingly the upper limit reaches the lower limit at approximately 9 memories simultaneously active in this short term memory system. This leads to the concept that the number of short term memory representations that can be kept simultaneously active may in practice be limited by (among other possible factors) the precision with which a biological system must tune *w*_inh_. With any number from 0 to 9 memories simultaneously active, the tolerance with which *w*_inh_ must be set is very fine. This leads us to suggest that at least in this model, 7 plus or minus 2 short term memories simultaneously active in a single attractor network may be a limit that is set at least in part by how finely in practice the inhibition needs to be tuned as the number of simultaneously active short term memories reaches 7 and above, and also by the sparseness of the representation, which in the cerebral cortex is not very sparse [34].

In order to investigate our hypothesis that synaptic facilitation can not only increase the number of memories that can be maintained simultaneously active, but may also make the system more robust with respect to its sensitivity to small changes in the parameters, we show the result in Fig. 4b of simulating the same short term memory network, but without synaptic facilitation. It is clear that not only is the capacity less without the effects of the synaptic facilitation used for Fig. 4a, but also for a given number of short term memories simultaneously active, the system without synaptic facilitation is more sensitive to small parameter changes, such as of *w*_inh_ as shown in Fig. 4b. In fact, it can be observed that Δ*w* = (*w*_inh_)_high_- (*w*_inh_)_low_ is larger for the same number of memories (greater than 2) when the system is endowed with synaptic facilitation. Thus the synaptic facilitation mechanism has the advantage that it also makes the system more robust, and thus more biologically plausible, as well as increasing the short term memory capacity. We emphasize that this type of robustness, relative insensitivity to the exact values of the parameters, is likely to be important in biological systems, in which specification of the properties of neurons and networks is not expected to be set with high precision.

### Mean Field Approach

**Figure 5:**
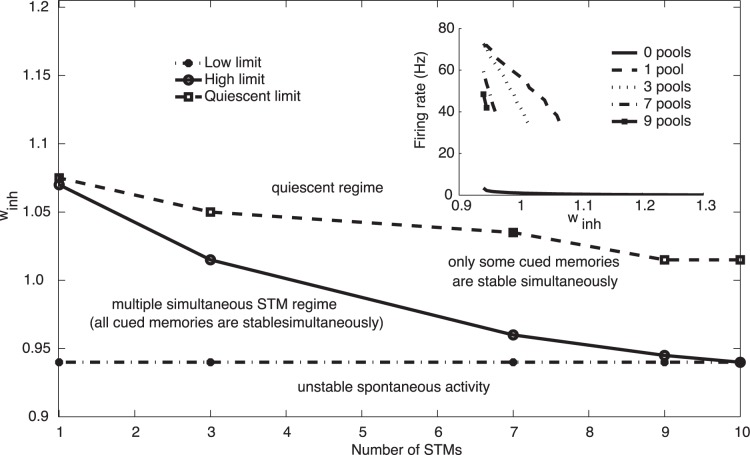
Mean field analysis showing the values of the inhibitory synaptic weights *w*_inh_ (between the lower and upper limits) within which different numbers of simultaneously active short term memories can be maintained stably. The different regimes are described in the text. There were 10 selective pools and the pattern of effective synaptic strengths (*w_+_*)_eff_ and (*w*_−_)_eff_ was determined from the corresponding spiking simulations. The coding level of the network was set to *a* = 0.1. The inset graph shows the firing rate of the subpopulations which have been stimulated as derived from the mean field analysis in the multiple active short term memory regime for different short term memory set sizes. For all the set sizes the firing rates obtained are physiologically plausible. The results displayed in this figure correspond to the results for the integrate-and-fire network shown in Fig. 4.

We complemented these integrate-and-fire simulations with mean field analyses to define the areas of the parameter space within which these multiple item short term memory effects were found, and to confirm by analytic methods the stability of the multiple short term memory system that we describe. The system analyzed with the mean field approach was equivalent to that described for the integrate-and-fire simulations [7], [35], that is it had 10 specific pools each with sparseness *a* = 0.1, one inhibitory pool, and, unless otherwise specified, the same synaptic weights as the integrate-and-fire simulations described (see Fig. 1, and the Methods). A novel aspect of the mean field implementation used was that we estimated the effective synaptic weights that resulted from the effects of the synaptic facilitation using the integrate-and-fire spiking simulations, and used those values for the effective synaptic weights in the modified mean field analyses described in detail in the methods. A hypothesis in which we were especially interested was that the parameter space for multiple short term memory became smaller, and therefore harder to prescribe when the network was built biologically, as the number 7±2 was exceeded. Of particular interest was the parameter *w*_inh_, which in previous work with this type of integrate-and-fire network in which one active pool was being investigated had the value 1.0 [46], [47]. In order to allow multiple memory representations to be active, *w*_inh_ was reduced to a lower value, typically 0.945, for the simulations described here. This reduction of inhibition was important in reducing the competition between the multiple active short term memory pools. If it was reduced too much, then the spontaneous state became unstable. If it was increased too much towards 1.0, then it was difficult to maintain active more than one pool of cued neurons. With the mean field analyses, we were able to show that the range of values of *w*_inh_, in which 3 or 7 multiple pools could be kept simultaneously active was smaller than when the requirement was just to maintain 1 pool active, as shown in Fig. 4a and 5. Moreover, with the modified mean field analyses it was possible to find a value of *w*_inh_ that allowed 9 out of the ten pools to remain simultaneously active (Fig. 4a and 5). The mean field approach thus allowed us to show analytically that the system we describe with synaptic facilitation could maintain up to 9/10 separate pools of neurons simultaneously active.

In more detail, for each *w*_inh_ value, a set of effective synaptic weights for (*w*_+_)_eff_ was obtained from the spiking simulations, and the modified mean field analysis was then performed on the resulting network. Table 2 shows the effective *w*_+_ for the cued pools, and for the non-cued pools. These values for (*w*_+_)_eff_ were obtained from the spiking simulations by multiplying the value of *w*_+_ by the value of *u*_∞_ that was obtained, and these values of (*w*_+_)_eff_ were used in the mean field analyses. *w*_inh_ was 0.945. The other values were the same as those used in the conventional mean field approach and in the spiking simulations shown in Fig. 2.

**Table 2:** Mean field analyses.

Number of pools	(*w*_+_)_eff_ cued	(*w*_+_)_eff_ noncued
0	0.97	0.97
1	2.21	1.2
3	2.21	0.97
7	2.16	0.67
9	2.14	0.62

The values for the effective synaptic weights (*w*_+_)_eff_ for the cued and the non-cued pools, for different numbers of cued pools as calculated from Equation 13.

Fig. 5 shows the results of the modified mean field analyses. The values for the parameter *w*_inh_ within which different operating regimes occur are shown for different numbers of cued short term memory pools. The first regime is with *w*_inh_ <0.94, in which case there is insufficent inhibition in the network, and some of the pools start firing even when no pools have been cued. This regime of unstable firing of the uncued state when the neurons should be firing at the spontaneous rate of approximately 3 Hz is labeled ‘unstable spontaneous activity’ in Fig. 5.

The second regime labeled ‘multiple simultaneous STM regime’ in Fig. 5 is the regime of interest in this paper. The region lies above the dashed line in Fig. 5 of unstable spontaneous activity and below the solid line. In this area in Fig. 5, any number of cued memories can be maintained stably. It is clear from Fig. 5 that for just one cued memory, the value for *w*_inh_ can be anywhere between 0.94 and 1.07. For any 3 of the 10 possible STMs to be stable when cued on, the value for *w*_inh_ can be anywhere between 0.94 and 1.02. For any 7 of the 10 possible STMs to be stable when cued on, the value for *w*_inh_ can be anywhere between 0.94 and 0.96. Thus *w*_inh_ must be set much more accurately when the required capacity is for any 7 of the 10 possible short term memory attractor states to be maintained stably. For any 9 of the 10 possible STMs to be stable when cued on, the value for *w*_inh_ must be 0.94 and nothing else will do. The mean field analysis thus confirms that it is possible to maintain 9/10 possible short term memory states active when the sparse of the representation is 0.1 with binary neurons. The useful operating region for multiple simultaneously active short term memories is thus between the low limit and high limit boundaries shown in Fig. 5 that are established by the modified mean field analyses. Previous mean field analyses have already demonstrated that without synaptic facilitation it is difficult to find a value for which more than 5 short term memories can be maintained simultaneously active [30], [31]. We have also replicated such findings (results not shown) in this study although, as discussed by Dempere-Marco et al. [31], when the traditional mean field approach is used, the role of the dynamics during the stimulation period must be carefully considered. The mean field analysis thus confirms that synaptic facilitation is an important mechanism by which the number of short term memories that can be maintained simultaneously active can be increased.

The third regime shown in Fig. 5 and labeled ‘only some cued memories are stable simultaneously’ refers to the case in which *w*_inh_ is above the solid line, and is so large that only a subset of the cued short term memory pools can maintain their activity. That is, the high firing rates expected of all cued pools cannot be maintained stably. Thus the multiple short term memory capability fails when *w*_inh_ increases to a value above the solid line in Fig. 5. The fourth regime shown in Fig. 5 is above the upper dashed line, when *w*_inh_ is so large that no cued pools maintain their high firing rates stably.

These effects of the synaptic facilitation on the performance of the short term memory network can be understood as follows. The synaptic facilitation has an effect similar to increasing the synaptic connection weights within each neural population that was activated by a cue on a particular trial, relative to the non-cued pools. Thus the effective synaptic weights of just the cued pools are increased for just that trial, and are very different from the synaptic weights of the uncued pools. In more detail, the effective synaptic weights within a pool that result from the synaptic facilitation are shown in Table 2, and indicate that when the neurons are firing fast, *u* approaches 1.0, and the effective synaptic weights for a cued pool become close to the value of 2.3 that was usually specified for *w*_+_. That value for *w*_+_ is sufficient to maintain a pool of neurons firing stably. On the other hand, for uncued pools, the value of *u* remains low, and the effect of this is to reduce (by the multiplicative effect on the synaptic weight) the effective synaptic weights to values that are below the value of approximately 2.1 needed to maintain a pool firing stably with a high rate in an attractor state. It is this that enables the network to remain stable using the mechanism of synaptic facilitation, with very many simultaneously active pools, in the face of the noise (stochastic fluctuations caused by the close to random firing times of the neurons and of the external inputs λ) in the system. In a network without synaptic facilitation, all the internal recurrent collateral synaptic weights of the pools (one pool for each stimulus) are of the same strength, so that any noise in the system may cause a jump from an active to an inactive pool as the weights and energy landscape [7], [48] for the different pools are rather similar. The synaptic facilitation makes the energy landscape have very deep basins just for the cued pools.

### The Short Term Memory Capacity with More Sparse Representations

**Figure 6:**
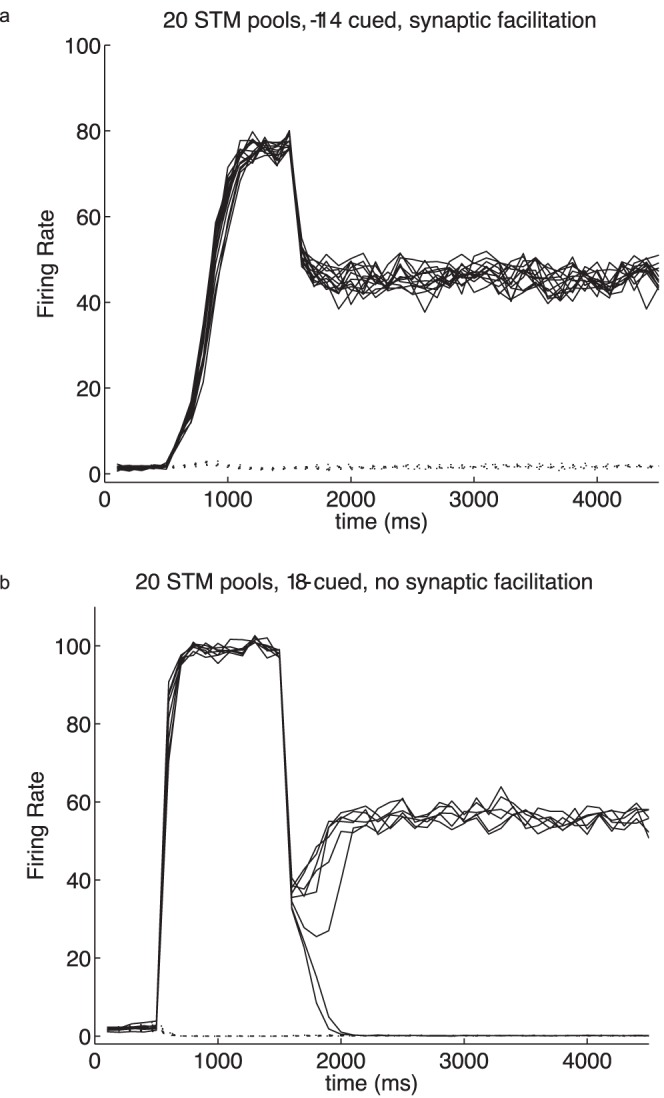
Simulations of short term memory with multiple simultaneously active pools with a sparseness of the representation *a* = 0.05. There were 20 pools in the integrate-and-fire simulations. (a) With synaptic facilitation, it was possible to cue on (500–1500 ms) up to 20 pools, and for all cued pools to remain stably active after the removal of the cues at 1500 ms. *w*_+_ = 3.5, *w*_inh_ = 0.95. In the example shown, 14 pools were cued, and all 14 remained firing in the short term memory period after the cues. The firing rates of all 20 pools are shown, with those within the cue set shown with solid lines, and those not cued with dotted lines. (b) Without synaptic facilitation, it was possible to cue on (500–1500 ms) up to 7 pools, and for these to remain stably active after the removal of the cues at 1500 ms. *w*_+_ = 2.5, *w*_inh_ = 0.975. In the example shown, 8 pools were cued, and only 6 remained firing in the short term memory period after the cues, with 2 of the pools not maintaining their firing rates. The firing rates of all 20 pools are shown, with those within the cue set shown with solid lines, and those not cued with dotted lines. There were 4000 neurons in these simulations, and λ_ext_ was 3.05 Hz per synapse on each of the 800 external synapses. Similar results to those in (b) could also be obtained with *w*_+_ = 6, *w*_inh_ = 1.1.

The sparseness of the representation, which for binary neurons is the proportion of the excitatory neurons active for any one stimulus, is another factor that we have found to be important in setting the capacity for the number of items that can be maintained simultaneously active in short term memory. So far in this paper, we have considered a network with a sparseness, *a*, of the representation of 0.1. The reason for choosing a representation that is not very sparse is that representations in the cerebral cortex are not very sparse, and 0.1 is a biologically realistic value for binary neurons to investigate, as shown by recordings from neurons in different cortical areas [34]. In fact, as representations in the cortex are graded, the measure of sparseness we have defined for the graded case indicates even less sparse representations than this [34]. We hypothesized that more sparse representations would enable more memories to be maintained active simultaneously, until the limit of all the excitatory neurons being active was approached. We tested the hypothesis by performing further simulations with 20 specific pools in the network each with the sparseness *a* = 0.05. We found that with synaptic facilitation incorporated as described here, it was possible to maintain 20 different short term memories active simultaneously (Fig. 6a, which shows 14 cued and perfect multiple short term memory) (*w*_+_ = 3.5; *w*_inh_ = 0.945). However, without synaptic facilitation, it was possible to maintain only seven of the 20 memories simultaneously active (Fig. 6b, which shows 8 cued, but only 6 maintained in short term memory, with two of the cued pools falling out of their high firing rate state) (*w*_+_ = 2.5; *w*_inh_ = 0.975). These results again indicate that the use of synaptic facilitation can greatly increase the number of short term memories that can be maintained simultaneously active. These results also show that there is no ‘magic’ limit on the number of memories that can be maintained simultaneously active in short term memory. The number is set in part by the sparseness of the representations, with sparse representations allowing more short term memories to be simultaneously active; and by the use of synaptic facilitation, which can increase the number of representations that can be kept simultaneously active, by effectively increasing the synaptic connection weights within each neural population that was activated by a cue on a particular trial, relative to the non-cued pools.

## Discussion

In this work, we have investigated what parameters appear to be important in setting the limit on the number of short-term memories that can be maintained active simultaneously in a discrete attractor network. A key parameter was found to be the inhibitory synaptic weights, which has led us to suggest that, at least in part, there may be a capacity limit set by how finely in practice the inhibition needs to be tuned in the network, and also by the sparseness of the representation. We have further shown that synaptic facilitation of the type found in the prefrontal cortex boosts such capacity limit by effectively increasing the synaptic strengths just for those pools to which a cue is applied, and then maintaining the synaptic facilitation by the continuing neuronal firing in just these pools when the cue is removed.

The neural mechanism described here for enabling a single attractor network to maintain multiple, in our case up to 9 with a sparseness *a* = 0.1, memories simultaneously active is very biologically plausible, in that recurrent collateral connections to nearby neurons with associative synaptic plasticity are a feature of the architecture of the neocortex [6], [33], as is synaptic facilitation. Indeed, synaptic facilitation is common in higher cortical areas such as the prefrontal cortex [41]–[43], in contrast with early sensory areas where depression is more usual [41]. This is very consistent with the evidence that the prefrontal cortex is involved in short-term memory [1], [6], [49], whereas it is important that in early sensory cortical areas the neurons faithfully represent the inputs, rather than reflecting short-term memory [6].

The use of synaptic facilitation in the multiple active short-term memory mechanism described is important to the success of the system, for without the synaptic facilitation it is difficult to maintain more than a few representations simultaneously active [29]–[31]. In the attractor network we used, we took into account the sparseness of the representation as shown here. In particular, with the sparseness *a* = 0.1 it was possible to maintain only 6 memories simultaneously active without synaptic facilitation, and 9 with synaptic facilitation in the same network. With *a* = 0.05 it was possible to maintain only 7 memories simultaneously active without synaptic facilitation, and 20 with synaptic facilitation. Thus synaptic facilitation greatly increases the number of memories that can be maintained simultaneously active, and this is one of the new findings described in this research. The use of synaptic facilitation is of conceptual interest in the mechanism described, for it is a non-associative process, which is what enables just the cued pools to remain active when they are cued without any further associative learning. Moreover, as shown here, the effect of the synaptic facilitation is sufficiently large that it can, in combination with the recurrent collateral connections, enable the process including the synaptic facilitation to be regenerated so that the short-term memory of just the cued pools can be maintained for many seconds.

To be clear, we note that the issue of how many memories can be maintained simultaneously active in a short-term memory is different from the issue of how many memories can be stored in an attractor network and later correctly retrieved one at a time, which is a much larger number that scales with the number of synapses on each neuron, can be increased by a reduction in the sparseness of the representation, is facilitated by diluted connectivity, and is in the order of thousands in a network with 10,000 recurrent collateral synapses onto every neuron [4], [6], [21], [50], [51].

As shown in Figs. 4 and 5, the precision with which the inhibition must be set increases as the number of memories required to be simultaneously active increases. In the simulation shown with 10 specific pools (*a* = 0.1), the limit was reached with 9 pools simultaneously active. In this case, 90% of the excitatory neurons were active. Under these circumstances, the constraint is to find a value of *w*_inh_ that is sufficiently small that all these cued pools can be active without quenching each other through the inhibitory neurons; and at the same time for *w*_inh_ to be sufficiently large that when no pools are cued, the spontaneous state is stable, a requirement for a short-term memory system.

This led us to hypothesize that if we reduced the sparseness of the representations, this would enable more representations to be maintained active simultaneously. We tested the hypotheses by performing further simulations with the sparseness *a* = 0.05, with 20 specific pools in the network. We found that with synaptic facilitation incorporated as described here, it was possible to maintain 20 different short-term memories active simultaneously (with an example of 14 simultaneously active illustrated in Fig. 6a). However, without synaptic facilitation, it was possible to maintain only seven of the 20 memories simultaneously active (Fig. 6b), and the increase in the number as the representations are made more sparse is consistent with mean field analyses [30]. These results show that there is no ‘magic’ limit on the number of memories that can be maintained simultaneously active in short-term memory. The number is set in part by the sparseness of the representations, with sparse representations allowing more short-term memories to be simultaneously active. However, the results show that there is a very real gain in the number that can be kept simultaneously active if synaptic facilitation is used as part of the mechanism.These points lead us to the following hypothesis. The number of memories that can be maintained simultaneously active is in practice in a single network in the cortex in the order of 7, because the sparseness of the representation is unlikely to be more sparse than *a* = 0.1. Representations that are somewhat distributed in this way are important to allow completion of incomplete representations in memory systems, and robustness against damage to synapses or neurons [6], [34]. In fact, as representations in the cortex are graded, the measure of sparseness we have defined for the graded case indicates even less sparse representations than the value of 0.1 [6], [34]. With that level of sparseness, a large number of neurons will be simultaneously active, and this will make setting the inhibition difficult, as just described. We are led therefore to the suggestion that the number of memories that can be kept simultaneously active in short term memory in a single cortical network is limited by the sparseness of the representation, which is not very sparse, and by the difficulty of setting the inhibition when many neurons are simultaneously active. Moreover, as we show here, synaptic facilitation can significantly increase the number that can be maintained simultaneously active, by effectively altering the energy landscape on each trial for just the pools of neurons that have been cued.

We note that these findings were obtained with non-overlapping pools of neurons, and that the restriction on the number that can be maintained simultaneously active will if anything be increased when there is overlap between the pools, due to interference between the different pools due to their overlap [29]. The system that we have simulated does in fact implement some functional overlap between the pools, through the effect of *w*_−_ (see Fig. 1), and this does simulate effects of interference and cross-talk between the different excitatory short term memory populations of neurons.

Another feature of the mechanism described is that it does not rely on oscillations, precise timing in the system, or a special mechanism to read out which short term memories have been cued: the firing rate of the cued neurons is available, and is the usual way that information is read out from memory [6], [34]. This is in contrast to another mechanism that has been proposed that is based on oscillations to implement multiple short term memories [52], [53]. Although there is some evidence that oscillations may play a role in short term memory function, there is controversy, with some studies indicating that there is an increase of power in the alpha band (9–12 Hz) [54] with short memory load whereas more recent studies indicate that an increase occurs in the lower range-theta (2–6 Hz) and gamma bands (28–40 Hz) with a power decrease in the alpha/beta band (10–18 Hz) (as discussed in Lundqvist et al. [55]) or in the theta band (4–12 Hz) [56]. Furthermore, the locus (or loci) of short term memory function leading to capacity limits have not been fully established. Thus, although the proposed model could be extended to account for oscillations by, for instance, changing the relative contribution of the slow NMDA and the fast AMPA receptors to the total synaptic currents [57], [58], or by introducing a mechanism based on the interplay of the different time constants of the synaptic facilitation and neuronal adaptation processes as in Mongillo et al. [38], we have preferred not to incorporate such mechanisms, and instead propose a minimal model that is investigated in depth in order to gain a fundamental understanding of how synaptic facilitation can boost short term memory capacity.

The mechanism described here may play an important part in language, by enabling a single cortical network of 1–2 mm^2^ to keep active multiple items simultaneously, representing for example individuals that are the subjects of a sentence [13], and thus reducing the load on the syntactic mechanisms that implement language in the brain. By syntax we mean in this context in computational neuroscience the ability to represent the fact that some sets of firing neurons might represent the subjects of a sentence, and other sets of firing neurons the objects in a sentence, so that some form of binding mechanism is needed to indicate which firing neurons are the subjects, and which are the objects. A further way in which the multiple short term memory mechanism described here may be useful in the brain’s implementation of language is that if there is a need to reactivate assemblies in a sentence that is being produced (for example to determine when forming a verb whether the subject was singular or plural), then non-specific activation working in conjunction with the remaining synaptic facilitation might enable those assemblies to be reactivated, as illustrated in Fig. 3.

Some approaches to short term memory consider continuous attractor networks in which the concept of the precision of the memory is relevant [27], but that concept does not apply to attractor networks with discrete representations where the term short term memory capacity, i.e. the number of separate memories that can be stored, actively maintained and correctly recalled, does not aim to reflect such precision. The reason for this is that, whereas continuous attractor networks account for precision by considering the dynamics of the width of the bumps that are actively maintained during the delay period [27], in discrete attractors such widths can not be defined. In a sense, discrete attractor networks can be considered a limit case of continuous attractor networks in which the items that can be encoded differ sufficiently from each other to engage clearly distinct populations of selective neurons. Discrete attractor networks are highly relevant when the items being stored are not part of a continuum, but are separate and different items [6], [59]. Thus, although most experimental (and theoretical) paradigms addressing the question of how neural resources are allocated to different items in short term memory [25]–[27] consider the accuracy dimension to discriminate between two main competing families of models (i.e. fixed capacity models *vs* dynamic allocation models), the use of saliency, an experimental variable that can be easily manipulated, has also demonstrated its value to contribute remarkable predictions. In particular, the predictions of a discrete attractor network similar to the one proposed in this work (although without synaptic facilitation) that was presented by the authors [31] lie somewhere between pure slot and pure shared resources models. This in agreement with the recent results by Wei et al. [27] obtained by making use of a continuous attractor network.

It is also worth noting that in the context of short term memory, the property investigated here is how many such discrete short term memory states can be maintained active simultaneously. While the number of memories that can be stored and correctly recalled is high (in the order of the number of synapses per neuron divided by the sparseness of the representation [6], [60]), the number that can be maintained simultaneously active is much smaller, with some of the relevant factors considered here.

In addition, we note that some types of short term memory encode the order of the items [52], [61], but that order information may not be a property of all types of short term memory, including for example that involved in remembering all the subjects in a sentence referred to above.

We further comment that the mechanism described here utilizes synaptic facilitation, and that this mechanism would enable items to remain active relative to other items even if at the same time there is some overall synaptic depression. Indeed, the important new issue that we address here is how synaptic facilitation can provide a mechanism for increasing the number of items that can be maintained simultaneously active in short term memory. We also note that long-term associative synaptic modification can occur rapidly, as shown by studies of long-term potentiation [6], so that the new attractor states required for new items to be stored in a short term memory could be set up rapidly.

Finally, we point out that factors that reduce synaptic facilitation [62] could cause a deterioration in short term memory by for example reducing effective synaptic weights, which in stochastic neurodynamical systems can decrease the stability of the high firing rate attractor states that implement short term memory [7], [63], and this opens a new avenue for helping to minimize the deterioration of short term memory that occurs in normal aging. We also note that given the potential role of the prefrontal cortex in the cognitive symptoms of schizophrenia [10], [12], factors that enhance excitatory synaptic connectivity such as synaptic facilitation, may provide useful approaches to explore for treatment [12], [64], [65].

## Supporting Information

Text S1 Supplementary Text. Conventional mean field approach.
(DOC)
