# Supplementary material for: Holding Multiple Items in Short Term Memory: A Neural Mechanism
Source: PLoS One. 2013 Apr 16;8(4):e61078. doi: 10.1371/journal.pone.0061078 (PMC3628858; doi:10.1371/journal.pone.0061078)
Supplement: Text S1 — Supplementary Text. Conventional mean field approach. (DOC) [file pone.0061078.s001.doc]

**Holding Multiple Items in Short Term Memory: A Neural Mechanism**

Edmund T. Rolls

Laura Dempere-Marco

Gustavo Deco

**Supporting Information**

*Text S1 Supplementary Text. Conventional mean field approach*

The details of the complete mean field analysis of the attractor neural network we investigate with conductance-based synaptic inputs can be found in Brunel and Wang . Following this approximation, the stationary dynamics of each neural population is described by the population transduction function. This provides the average population rates after a period of dynamical transients as a function of the average input current. Such stationary rates can be found as the stable solutions of a self-consistency condition and correspond to the stable attractors of the structured network.

In this formulation the potential of a neuron is calculated as:

| $\tau_{x}\frac{dV(t)}{dt}=-V(t)+\mu_{x}+\sigma_{x}\sqrt{\tau_{x}}\eta(t)$ | (S.1) |
| --- | --- |

where *V*(*t*) is the membrane potential, *x* labels the populations, *τ_x_* is the effective membrane time constant, *μ_x_* is the mean value the membrane potential would have in the absence of spiking and fluctuations, *σ_x_* measures the magnitude of the fluctuations and *η* is a Gaussian process with absolute exponentially decaying correlation function with time constant *τ_AMPA_*. The quantities *μ_x_* and σ*_x_* are given by:

| $\mu_{x}=\frac{(T_{ext}\nu_{ext}+T_{AMPA}n_{x}^{AMPA}+\rho_{1}n_{x}^{NMDA})V_{E}+\rho_{2}n_{x}^{NMDA}\left\langle V\right\rangle+T_{I}n_{x}^{GABA}V_{I}+V_{L}}{S_{x}}$ | (S.2) |
| --- | --- |
| $\sigma_{x}^{2}=\frac{g_{AMPA,ext}^{2}(\left\langle V\right\rangle-V_{E})^{2}N_{ext}\nu_{ext}\tau_{AMPA}^{2}\tau_{x}}{g_{m}^{2}\tau_{m}^{2}}$ | (S.3) |

In these equations *ν_ext_* corresponds to the external incoming spiking rate, *ν_I_* is the spiking rate of the inhibitory population, and τ*_m_* = *C_m_*/ *g_m_* (with appropriate values for the excitatory or inhibitory cells, provided in Table 1). The rest of variables are given by:

| $S_{x}=1+T_{ext}\nu_{ext}+T_{AMPA}n_{x}^{AMPA}+(\rho_{1}+\rho_{2})n_{x}^{NMDA}+T_{I}n_{x}^{GABA}$ | (S.4) |
| --- | --- |
| $\tau_{x}=\frac{C_{m}}{g_{m}S_{x}}$ | (S.5) |
|  |  |
| $n_{x}^{AMPA}=\sum_{j=1}^{p} r_{j}w_{jx}^{AMPA}\nu_{j}$ | (S.6) |
| $n_{x}^{NMDA}=\sum_{j=1}^{p} r_{j}w_{jx}^{NMDA}\psi(\nu_{j})$ | (S.7) |
| $n_{x}^{GABA}=\sum_{j=1}^{p} r_{j}w_{jx}^{GABA}\nu_{j}$ | (S.8) |
| $\psi(\nu)=\frac{\nu\tau_{NMDA}}{1+\nu\tau_{NMDA}}\left(1+\frac{1}{1+\nu\tau_{NMDA}}\sum_{n=1}^{\infty} \frac{\left(-\alpha\tau_{NMDA,rise}\right)^{n}T_{n}\left(\nu\right)}{\left(n+1\right)!}\right)$ | (S.9) |
| $T_{n}\left(\nu\right)=\sum_{k=0}^{n} \left(-1\right)^{k}\left(\begin{matrix} n \\ k \end{matrix}\right)\frac{\tau_{NMDA,rise}\left(1+\nu\tau_{NMDA}\right)}{\tau_{NMDA,rise}\left(1+\nu\tau_{NMDA}\right)+k\tau_{NMDA,decay}}$ | (S.10) |
| $\tau_{NMDA}=\alpha\tau_{NMDA,rise}\tau_{NMDA,decay}$ | (S.11) |
| $T_{ext}=\frac{g_{AMPA,ext}\tau_{AMPA}}{g_{m}}$ | (S.12) |
| $T_{AMPA}=\frac{g_{AMPA,rec}N_{E}\tau_{AMPA}}{g_{m}}$ | (S.13) |
| $\rho_{1}=\frac{g_{NMDA}N_{E}}{g_{m}J}$ | (S.14) |
| $\rho_{2}=\beta\frac{g_{NMDA}N_{E}\left(\left\langle V_{x}\right\rangle-V_{E}\right)\left(J-1\right)}{g_{m}J^{2}}$ | (S.15) |
| $J=1+\gamma\exp\left(-\beta\left\langle V_{x}\right\rangle\right)$ | (S.16) |
| $T_{I}=\frac{g_{GABA}N_{I}\tau_{GABA}}{g_{m}}$ | (S.17) |
| $\left\langle V_{x}\right\rangle=\mu_{x}-\left(V_{thr}-V_{reset}\right)\nu_{x}\tau_{x},$ | (S.18) |

where *p* denotes the number of selective excitatory subpopulations (i.e. ten in these mean field analyses), *r_x_* is the coding level or fraction of neurons in each excitatory subpopulation *x* (and is the same as *a* the sparseness of the representation described in the paper which is more commonly used in neurophysiological investigations ), *w_jx_* is the synaptic weight of the connections from population *x* to population *j*, *ν_x_* is the spiking rate of the excitatory subpopulation *x*, γ = [Mg^++^]/3.57, β = 0.062, and the average membrane potential $\left\langle V_{x}\right\rangle$ has a value in the range [-55,-50] mV.

In the conventional mean field approach, a set of fixed synaptic weights is used, which establish the strength of the different connections between all the subpopulations. These weights are normally obtained in accordance with the hypothesis of Hebbian associative plasticity, i.e. synaptic efficacies are modified by neural activity during a training process through long-term potentiation (LTP) and long-term depression (LTD). It is assumed that these synaptic weights have been set through repeated presentations of *p* different stimuli in random sequences. This scenario leads to the recurrent connections between cells within the same selective subpopulation becoming strengthened to reach a value *w*_+_, which is *w*_+_ > 1, where 1 is the baseline synaptic connectivity strength between populations, while connections between cells from different selective subpopulations are weakened to assume a value *w* −, where 0 < *w*_−_ < 1. In this study, in those cases when *w_jx_^GABA^* is different from zero, we specify whether it takes the value *w*_inh_ (the value of the inhibitory to excitatory connections), or the value *w* = 1 (the value of the inhibitory to inhibitory connections). In addition, *w_jx_*^AMPA^ = *w_jx_^NMDA^*, and this may take the values *w_+_*, *w-* or *w* = 1, depending on the neural populations *x* and *j* which are connected.

The average firing rates of the different populations in the network can be calculated in the mean field approximation from the solution of a set of *n* nonlinear equations which depend on *μ_x_* and σ*_x_* as follows:

| $\nu_{x}=\varphi\left(\mu_{x},\sigma_{x}\right)$, *x* = 1, ... *n* | (S.19) |
| --- | --- |

where $\varphi$ is the transduction (or activation) function of population *x* (i.e. the *f*-*I,* or rate vs current, curve), which enables the calculation of the output rate of population *x* in terms of its input currents, which in turn depend on the rates of all the other neural populations communicated through the synaptic weights. The transduction function $\varphi$ is given by:

| $\varphi(\mu_{x},\sigma_{x})=\left(\tau_{rp}+\tau_{x}\int_{\beta(\mu_{x},\text{ }\sigma_{x})}^{\alpha(\mu_{x},\text{ }\sigma_{x})} du\sqrt{\pi}\exp(u^{2})\left[1+\text{erf}(u)\right]\right)^{-1}$ | (S.20) |
| --- | --- |
| $\alpha(\mu_{x},\sigma_{x})=\frac{\left(V_{thr}-\mu_{x}\right)}{\sigma_{x}}\left(1+0.5\frac{\tau_{AMPA}}{\tau_{x}}\right)+1.03\sqrt{\frac{\tau_{AMPA}}{\tau_{x}}}-0.5\frac{\tau_{AMPA}}{\tau_{x}}$ | (S.21) |
| $\beta(\mu_{x},\sigma_{x})=\frac{\left(V_{reset}-\mu_{x}\right)}{\sigma_{x}}$ | (S.22) |

with erf(*u*) the error function and *τ_rp_* the refractory period which is taken as 2 ms for excitatory neurons and 1 ms for inhibitory neurons. To solve the equations defined by (S.19) for all *x,* we integrate numerically (S.23), whose fixed points are solutions of equation (S.19):

| $\tau_{x}\frac{d\nu_{x}}{dt}=-\nu_{x}+\varphi\left(\mu_{x},\sigma_{x}\right)$ | (S.23) |
| --- | --- |

The network dynamics could converge to different attractor states corresponding to multi-item memory states or *m*-memory states, with *m* being the number of coactivated excitatory subpopulations of neurons, each one of which represents a memory.

References

1. Brunel N, Wang XJ (2001) Effects of neuromodulation in a cortical network model of object working memory dominated by recurrent inhibition. Journal of Computational Neuroscience 11: 63-85.

2. Rolls ET, Treves A (2011) The neuronal encoding of information in the brain. Progress in Neurobiology 95: 448-490.
